# CoCStom trial: study protocol for a randomised trial comparing completeness of adjuvant chemotherapy after early versus late diverting stoma closure in low anterior resection for rectal cancer

**DOI:** 10.1186/s12885-015-1838-0

**Published:** 2015-11-21

**Authors:** Flavius Sandra-Petrescu, Florian Herrle, Axel Hinke, Inga Rossion, Heiko Suelberg, Stefan Post, Ralf-Dieter Hofheinz, Peter Kienle

**Affiliations:** Surgical Department, University Medical Centre Mannheim, Medical Faculty Mannheim, University of Heidelberg, Theodor-Kutzer-Ufer 1-3, 68167 Mannheim, Germany; Wissenschaftlicher Service Pharma GmbH, Karl-Benz-Str. 1, 40764 Langenfeld, Germany; Study Center of the German Surgical Society, Im Neuenheimer Feld 110, 69120 Heidelberg, Germany; Oncological Department, University Medical Centre Mannheim, Medical Faculty Mannheim, University of Heidelberg, Theodor-Kutzer-Ufer 1-3, 68167 Mannheim, Germany

**Keywords:** Early stoma closure, Completeness of adjuvant chemotherapy, Rectal cancer, Low anterior resection with total mesorectal excision

## Abstract

**Background:**

Current evidence supports a diverting stoma in patients undergoing low anterior resection with total mesorectal excision for rectal cancer as it reduces clinical severity of anastomotic leakage. However, relevant stoma morbidity after rectal cancer surgery exists and has a significant impact on quality of life. Moreover, a diverting stoma has an influence on completeness of chemotherapy but it remains unclear in which way. There is no evidence regarding optimal timing for stoma closure in relation to adjuvant chemotherapy. Two randomised controlled trials have studied early stoma closure after low anterior resection in patients with rectal cancer, one of them showing that early closure around day 8 after resection is possible without increasing morbidity.

**Methods/Design:**

CoCStom is a randomised multicentre trial comparing completeness of adjuvant chemotherapy as primary endpoint after early (8–10 days after resection, before starting adjuvant therapy) versus late (~26 weeks after resection and completion of adjuvant therapy) stoma closure in patients with locally advanced rectal cancer undergoing low anterior resection after neoadjuvant therapy. After exclusion of post-operative anastomotic leakage 257 patients from 30 German hospitals are planned to be included in order to assure a power of 80 % for the confirmatory analysis of at least 214 evaluable cases. An absolute increase of 20 % for the rate of completely administered adjuvant chemotherapy is regarded as a clinically meaningful step forward and serves as basis for sample size calculation. Quality of life, stoma-related complications, individual completeness of chemotherapy rate, percentage of patients stopping adjuvant therapy or undergoing dose modifications or delay, oncological outcomes, cumulative days of hospitalisation and number of readmissions, rate of symptomatic anastomotic leaks after stoma closure, mortality, post-operative complications and toxicity of adjuvant chemotherapy are secondary endpoints.

**Discussion:**

The CoCStom trial aims to clarify optimal timing of stoma closure in the context of adjuvant chemotherapy. Depending on the results of the trial, patients could benefit either from early or late stoma closure in regard to long term oncological survival due to a higher rate of completeness of adjuvant chemotherapy treatment and thus better effectiveness.

**Trial registration:**

German Clinical Trials Register, DRKS00005113. Registered 28 August 2013

**Electronic supplementary material:**

The online version of this article (doi:10.1186/s12885-015-1838-0) contains supplementary material, which is available to authorized users.

## Background

Low anterior resection (LAR) is the procedure of choice for rectal cancer of the middle and lower third [1, 2]. An anastomosis performed close to the pelvic floor is associated with a relevant risk of anastomotic leakage (3–17 %), which contributes to mortality [3]. Based on level-1 evidence a diverting stoma effectively reduces the rate of symptomatic anastomotic leakage [4].

However, a diverting stoma also causes morbidity, which may affect nearly half of all patients as shown in an analysis of patients with protective stoma for distal colonic anastomosis [5]. Stoma-related morbidity includes high-output stoma, stoma prolapse, small bowel obstruction and wound infection and has been evaluated in several studies [5–7]. Moreover, a stoma may impair quality of life (QoL). The global QoL as well as the physical functioning and role scores are negatively influenced by stoma after LAR with total mesorectal excision (TME) as shown in two prospective observational longitudinal studies of 22 [8] and 24 patients [9] respectively. These scores and additionally the mental health improved 6 weeks after ileostomy closure. Contrarily, 23 patients receiving a high anterior resection without stoma had higher QoL scores postoperatively compared to preoperatively [9].

Very little evidence regarding the optimal timing for stoma closure was found despite an extensive systematic search in relevant literature databases and trial registries. Our prospective multicentre pilot study investigating the timing of stoma closure and longitudinal QoL before LAR with TME, before stoma closure and 6 months after stoma closure on 171 patients from 17 participating German surgical centres showed that this is in median performed 5.1 months after creation, with a wide range (0.6–32 months) [10].

Earlier stoma closure is feasible with low morbidity and no mortality as a pilot study demonstrated by closing the stoma 11 days after tumour resection [11]. Finally, a prospectively randomised multicentre trial with 186 patients confirmed that stoma closure on day 8, during the same hospitalisation, is possible without increase of the perioperative complication rate. In this study, which reflects the best available evidence, the overall postoperative complication rate was significantly lower in the early closure group (small bowel obstruction early vs. late: 3 vs. 16 %, *p* = 0.002 and medical complications: 5 vs. 15 %, *p* = 0.021) [6]. The hospitalisation period was also significantly shorter in the early stoma closure group (*p* = 0.013) but there was no difference in QoL (*p* = 0.566). Major drawback of this study is that there is no data on administered adjuvant chemotherapy. The only larger, albeit retrospective, study investigating stoma related complications and postoperative morbidity in 120 patients with colorectal carcinoma undergoing colorectal resections in regard to adjuvant chemotherapy showed a trend toward fewer complications (12.5 %) if stoma closure was performed early (i.e. before starting adjuvant chemotherapy) rather then during (42.9 %) or after (21.2 %) chemotherapy [7].

In contrast, an early closure of the stoma may also negatively influence the completeness of chemotherapy (CoC) due to complications like symptomatic anastomotic leakage or LAR syndrome. The latter is a well-known clinical condition occurring after deep anterior rectal resection potentially severely impairing QoL in these patients [12, 13]. This usually becomes evident during the first months after stoma reversal and may also aggravate side-effects of chemotherapy, again resulting in less patients receiving all cycles of planned chemotherapy.

Timing of diverting stoma closure in advanced rectal cancer has also not yet been adequately investigated in the setting of adjuvant chemotherapy. Moreover, no randomised studies specifically investigating the influence of CoC on overall survival (OS) in patients having undergone rectal resection for rectal cancer have yet been published. We re-analyzed the data from our phase-III study comparing 5-FU and capecitabine in the perioperative treatment of patients with stage II and III rectal cancer with regard to influence of CoC on survival [14]. CoC in this trial was associated with a significant improvement of 3-year disease free survival (DFS) as well as 5-year OS. Therefore, since adjuvant chemotherapy is considered to improve long-term survival in nodal-positive (stage III) disease and recommended as standard treatment in the German S3-guidelines [15, 16] it is suitable to consider CoC as primary endpoint in a prospective randomised clinical trial. Moreover, in order to improve the rates of completed adjuvant chemotherapy treatment in rectal cancer, it is highly patient relevant to investigate the influence of a stoma.

In conclusion, the optimal timing for stoma closure with regard to CoC remains controversial. It seems advantageous to close the stoma as early as possible, but on the other hand early closure may result in more complications which may impair the administration of all planned cycles of adjuvant chemotherapy.

## Methods/Design

### Trial design

CoCStom is an investigator-initiated prospective randomised open-label multicentre trial with two parallel study groups.

### Patient population

Patients with rectal cancer stadium UICC II-III undergoing low anterior resection with diverting stoma after neoadjuvant therapy and planned adjuvant chemotherapy will be included in the trial. A detailed overview of all eligibility criteria is given in Table 1.

**Table 1:** Inclusion and exclusion criteria

Inclusion criteria	Exclusion criteria
▪ Temporary diverting stoma (independent from the stoma type)	▪ ASA >3
▪ Elective curative LAR with TME (laparoscopic, open or converted) after neoadjuvant therapy (long course chemoradiation or short-term radiotherapy - 5 × 5 Gy) for UICC II-III rectal cancer	▪ Inflammatory bowel disease
▪ No anastomotic leakage (endoscopic or contrast enema assessment of the anastomosis around day 7 after LAR)	▪ Contraindication to adjuvant chemotherapy arising after rectal cancer resection [15]
▪ Indication to undergo adjuvant chemotherapy (according to current German guidelines the pre-therapeutic stage serves as basis for the adjuvant treatment decision) [15]	▪ Disease progress to UICC IV under neoadjuvant therapy
▪ Patient has given written informed consent	▪ Immunocompromised patients (HIV-positive, patients currently under chemotherapy for other diseases or patients under immunosuppressive therapy, e.g. Prednisolone >10 mg)
▪ Age ≥18 years	▪ Participation in another intervention-trial with interference of intervention and outcome of this study
▪ Patient is able to cooperate (ability of subject to understand character and individual consequences of the clinical trial)	

*HIV* human immunodeficiency virus

### Scheme of intervention

Only centres with a special focus on colorectal surgery performing at least 20 LARs per year for rectal cancer (expertise based trial) in accordance to the German guidelines (e.g. adherence to the principles of total mesorectal excision) can take part in the CoCStom trial. This corresponds to certification requirements for colorectal cancer centres of the German Cancer Society [17].

In order to minimise performance bias, LAR within the study is performed only by surgeons with a minimum experience of 20 LARs for cancer (‘life-time experience’). In addition, surgeons need to have an adequate experience in performing the investigated stoma reversal approach. The recommended minimum number of stoma closures is 10. The individual surgeon’s experience will be documented. Each study centre provides at least one surgeon meeting these requirements. Stoma closure will be performed, according to the centre standard of care, before (experimental) or after completion (standard) of adjuvant chemotherapy.

The adjuvant chemotherapy will be performed according to the S3-guidelines for colorectal cancer [15] and recent clinical evidence [14, 18, 19] using 5-fluorouracil- (5-FU) and oxaliplatin-based regimes.

### Recruitment and trial timeline

**Fig. 1:**
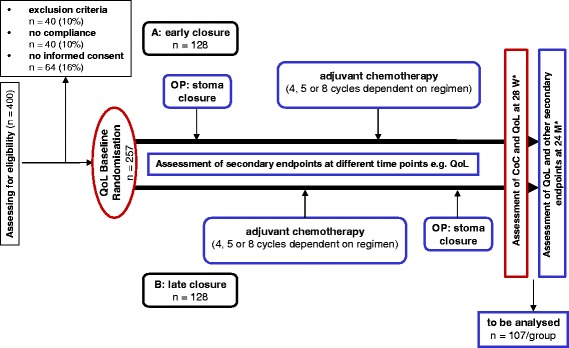
Expected patient flow from screening to final analysis. Early closure group: stoma closure and first chemotherapy (CTx) will be done around 2 days and within 4 (earliest) to 12 (latest) weeks respectively after randomisation. Late closure group: first CTx will be done within 4 (earliest) to 12 (latest) weeks after randomisation; stoma closure will be done 4 weeks after end of CTx. Randomisation will be performed around day 7 after LAR (after exclusion of an anastomotic leakage). To reach the statistically calculated goal of 257 recruited patients, at least 128 patients have to be randomised into each trial arm. W: weeks, M: months, OP: operation, *: after randomisation

Recruitment of the patients has started in December 2013. The duration of the trial for each patient is expected to be 24 months including follow-up at 7 and 24 months after randomisation. The duration of the entire trial is expected to be 69 months (Fig. 1).

### Assignment of intervention and randomisation

Patients should be screened as soon as the tumour stage (UICC II-III) is confirmed and the neoadjuvant therapy (chemoradiation or 5×5 Gy schedule) has been defined. All patients having undergone neoadjuvant therapy for clinically staged cT3/4 Nx or cTx N+ rectal cancer (staged with endorectal ultrasonography + CT or endorectal ultrasonography + MRI), LAR with TME and protective stoma and after having given written informed consent will be registered for the trial. The randomisation will take place around 7 days after LAR by central online registration, after exclusion of anastomotic leakage by endoscopy and/or radiographic enema. Patients will be allocated to two groups, early group (experimental) - stoma closure between day 8 and 10 after LAR before starting the adjuvant chemotherapy and late group (standard) - stoma closure after adjuvant chemotherapy around week 26 after LAR. A central computer-generated block-randomisation with alternating block sizes will be performed by web-randomisation within the electronic case report forms (eCRF) system in order to ensure an adequate random allocation. There will be stratification by centre.

### Interventions and trial flow

The stoma may be closed either by a direct suture of the anterior wall or by an end-to-end, side-to-side or end-to-side anastomosis after a segmental resection of the bowel (stapler or hand suture) according to the centre’s standard of care.

The following 5-FU- and oxaliplatin-based adjuvant chemotherapy schedules are allowed:

- four cycles of bolus 5-FU 500 mg/m^2^ (days 1–5, repeated at day 29) or bolus 5-FU 350 mg/m^2^ plus folinic acid 20 mg/m^2^ (days 1–5, repeated at day 29),
- five cycles of capecitabine 2500 mg/m^2^ (days 1–14, repeated at day 22),
- XELOX-Schedule – five cycles of capecitabine 2000 mg/m^2^ (days 1–14, repeated at day 22) plus oxaliplatin 130 mg/m^2^ (day 1, repeated at day 22),
- De Gramont-Schedule – eight cycles of bolus 5-FU 400 mg/m^2^ (day 1, repeated at day 15) plus 5-FU 600 mg/m^2^ over 24 h (days 1 and 2, repeated at day 15) plus folinic acid 200 mg/m^2^ (day 1, repeated at day 15),
- FOLFOX6-Schedule – eight cycles of bolus 5-FU 400 mg/m^2^ (day 1, repeated at day 15) plus 5-FU 2400 mg/m^2^ over 46 h (day 1, repeated at day 15) plus folinic acid 400 mg/m^2^ (day 1, repeated day 15) plus oxaliplatin 85 mg/m^2^ (day 1, repeated at day 15).

In both arms, adjuvant chemotherapy starts within 4 (earliest) and 12 (latest) weeks after randomisation, unless unexpected complications occur. Each participating institution should preferably select one of the regimens for their patients, before randomisation, in order to avoid bias. When a regimen has been started in an individual patient, this regimen should be continued throughout the study period. Administration of chemotherapy and regular blood tests as well as the treatment of adverse events (AEs) will be done according to the standard operating procedure implemented in the various oncological departments. Concomitant therapies (e.g. antiemetics) are allowed in accordance to local standard treatment but dose reduction should be done according to the protocol and to NCI-CTCAE criteria v.4.03 [20] unless it is not in the best interest of the treated patient (Table 2). Concurrent administration of any other anti-cancer therapy is not permitted during trial participation. The treatment and prophylaxis of some AEs such as neuropathy [21], hand-foot skin reaction [22], antiemetic treatment [23], chemotherapy induced diarrohea [24], and high-output stoma [25] should be performed according to the international standards.

**Table 2:** Dose modification scheme adapted to severity of adverse events

	Grade 2	Grade 3	Grade 4
1. occurrence	Withold therapy until AE has resolved to grade 0–1.	Withold therapy until AE has resolved to grade 0–1.	Withold therapy until AE has resolved to grade 0–1.
	Continue with 100 % of the starting dose.	Continue with 75 % of the starting dose	Continue with 50 % of the starting dose
2. occurrence	Withold therapy until AE has resolved to grade 0–1.	Withold therapy until AE has resolved to grade 0–1.	Stop treatment.
	Continue with 75 % of the starting dose.	Continue with 50 % of the starting dose.	
3. occurrence	Stop treatment	Stop treatment	

### Description of the trial visits

Trial visits will be performed in the corresponding clinical centres (surgical and oncological departments). Examples for trial visits for chemotherapy regimen with eight cycles are given as Additional file 1 and Additional file 2.

### Outcome measures and definitions

CoC is defined as primary endpoint and can be assessed objectively immediately after completion of treatment. CoC will be measured 28 weeks after randomisation. This point of time reflects the status after planned complete chemotherapy administration in both trial groups and after the stoma has already been closed.

CoC will be assessed by determining the proportion of the randomised patients which complete all planned cycles of adjuvant chemotherapy. Secondary endpoints and their definition are presented in Additional file 3.

### Data management

Data must be reported on eCRF. All protocol-required information collected during the trial must be entered by the investigator or authorised staff members as soon as possible after information is collected. Any outstanding entries must be completed immediately after the final examination. It is the responsibility of the investigator to review and sign the eCRFs.

### Compliance/rate of loss to follow-up

In order to maximise the number of participating patients to the study, disabled patients may be visited by a “flying study nurse”. Phone interviews are only exceptionally allowed.

A rate of 10–20 % of drop-outs and losses to follow-up are considered in order to have a sufficient power in the per-protocol analysis.

### Safety assessments and reporting of adverse events

Analysis of safety-related data is performed by calculation and descriptive comparison of the rates of AEs and serious adverse events (SAEs) based on all patients receiving at least one protocol-specified procedure.

An AE is defined as any untoward medical event in a patient which occurs following surgery or chemotherapy. A SAE is defined as any AE that results in death, immediately life threatening event, hospitalisation or prolongation of hospitalisation, persistent or significant disability or incapacity. SAEs will be classified according to intensity (mild, moderate, severe, life-threatening, death), outcome (recovered completely, recovered with sequelae, not recovered with or without therapy of SAE, death, unknown), and causality (doubtful, possibly related, probably related, definitely related, not assessable).

The investigator must report every event meeting the protocol definition of an SAE to the coordinating investigator until the next working day after having become aware of the event.

### Statistical methods

#### Sample size calculation

The sample size calculation is based on the analysis of the primary endpoint, completeness of adjuvant chemotherapy. According to our recent randomised phase III trial on perioperative treatment of stage II and III rectal cancer, a total of 61.7 % (capecitabine) and 57.5 % (5-FU), respectively, started adjuvant chemotherapy, and 45.7 and 40.0 % had their scheduled cycles completed [14]. These data are consistent with the findings in other studies investigating perioperative chemo (radio)therapy in rectal cancer.

Based on this data, we assume a CoC rate of 42 % for the standard patient group with a diverting stoma during adjuvant chemotherapy. A 20 % absolute increase of CoC (42–62 %) is regarded as a clinically meaningful step forward. In order to significantly detect this difference on a type I error level of 5 % (two-sided) with a power of 80 %, a sample size of 107 is required in each arm based on the application of Fisher’s exact test. The Mantel-Haenszel procedure stratifying for centre will be used for the primary hypothesis of the trial.

Any major deviation from these CoC rate assumptions will lead to increased power, if the delta of 20 % is retained. According to the primary endpoint, the number of losses in the intention-to-treat (ITT) population should be minimal. A rate of 10–20 % of drop-outs and losses to follow-up is expected. Thus, about 260 patients should be randomised to ascertain sufficient power in the per-protocol analysis. Stratification by centre is foreseen, since centre-specific characteristics such as the quality of surgery, patient guidance with respect to chemotherapy compliance, etc. may have a major influence on important outcome parameters.

#### Definition of analysis population

Patients who were enrolled although they unequivocally did not fulfil the selection criteria of the trial a priori (“non-eligible”) will be excluded from the statistical analysis, in accordance with ICH recommendations [26].

All other patients will primarily be evaluated in an ITT analysis according to allocation by randomisation. A second analysis (“per protocol”) of the primary endpoint will include only patients with full documentation on the amount of adjuvant chemotherapy received (i.e. including patients with a reliable documentation of no or incomplete adjuvant treatment).

All patients having received at least one application of protocol-defined therapy are evaluable for safety.

#### Analysis variables and statistical methods

- Primary analysis

The character of the primary analysis is confirmatory. The analysis is based on the ITT population using the ITT principles (dropouts and missing data will be counted as failures). For the analysis of the primary endpoint, the Mantel-Haenszel stratified test will be used. Corresponding 95 % confidence interval (CI) will be calculated for the CoC rates in both groups and for the rate difference and odds ratio (OR).

- Sensitivity analysis

The primary endpoint will be additionally analysed based on the per-protocol set comprising all patients with full documentation on the amount of adjuvant chemotherapy received. A multivariate analysis (logistic regression model) will be applied to account for the effect of potential prognostic factors such as for example age, performance status, location of the primary tumour, and T-stage.

- Secondary analysis

All secondary analyses have only exploratory character. P-values may only be used descriptively. All variables will be described at least by the number of observations, mean, standard deviation, median, minimum and maximum, or counts/percentages, as appropriate.

QoL data will be scored according to the algorithm described in the EORTC QLQ-C30 scoring manual [27]. While data on all functional and symptoms scales will be described, formal comparisons are going to focus on global QoL and symptoms related to the impact of chemotherapy and surgery on rectal cancer patients specifically (overall QoL, diarrhoea, constipation, vomitting).

In case of disease-free survival, local recurrence-free survival and distant recurrence-free survival Kaplan Meier survival curves for the groups will be presented and compared by the log rank test. Further secondary endpoints will be compared using a Wilcoxon-Mann–Whitney-U test or tests for contingency tables, as appropriate according to the type of data.

No interim analysis is planned. However, while the main statistical analysis is performed, as soon as documentation of post-operative therapy is completed in all patients at about 7 months post randomisation, a second analysis on long-term endpoints is performed about 1.5 years later.

### Individual trial termination

Patients may be withdrawn from the trial any time at their own request without giving reasons for their decision or if, in the investigator’s opinion, continuation of the trial would be detrimental to the patient’s well-being.

### Premature closure of the trial

The trial may be prematurely closed by the principal investigator in consultation with the steering committee. If the termination of the trial becomes necessary, the steering committee will discuss this issue with the independent data safety and monitoring board (DSMB). Reasons that may necessitate a termination of the trial include a potential health hazard caused by the trial treatment indicated by incidence or severity of SAEs in this trial, unsatisfactory patients’ enrolment, severely inaccurate and/or incomplete data recording or external evidence demanding a termination of the trial. The Ethic Committees will be informed.

### Trial organization and administration

#### Monitoring

All participating centres were personally trained and introduced into study-specific procedures during initiation visit. Regular on-site monitoring visits are planned at all sites. For at least 5 % of all subjects a 100 % clinical source data verification (SDV) for all clinical items is planned. The extent of further SDV and/or the frequency of monitoring visits will be adapted for individual centres depending on the quality of data or if common protocol violations are observed. In addition to the SOPs and to the trial protocol, procedures will be predefined in a study-specific monitoring manual and an eCRF manual. Queries will be issued by the monitor as well as the data manager and have to be answered by the investigators within a short time to avoid errors in data capture or entry being carried forward.

#### Steering committee

The steering committee supervises the conduct of the trial and issues recommendations for early termination, modifications or continuation of the trial, if necessary. It comprises seven members (three surgeons, two oncologists, radio-oncologist and statistician).

#### Data and safety monitoring board (DSMB)

To enable an independent risk assessment for treatment, potentially related SAEs will be noted and periodically (at least once a year during the first 24 months) assessed by the independent DSMB. Clinical monitoring will supervise patient’s safety and integrity of the clinical data. DSMB is an independent committee of 3 members (a surgeon, a radio-oncologist, and a statistician) whose task is to review the status of the clinical trial and make recommendations to the clinical research group concerning trial conduct, modification and/or publication. DSMB members will be asked to give advice on whether the accumulated safety data − notably SAEs − from the trial, together with results from other relevant trials, justify continuing recruitment of further patients. A decision to discontinue recruitment, in all patients or in selected subgroups, will be made only after discussion of the issue with the steering committee.

#### Ethical and legal aspects

The trial protocol, informed consent document and all trial specific documents were approved by the Ethics Committee II of the Medical Faculty of Mannheim, University of Heidelberg. Additionally, an ethical approval has been obtained at all participating centres (Additional file 4). The responsible investigator will ensure that this study is conducted in agreement with the Declaration of Helsinki (Tokyo, Venice, Hong Kong, Somerset West, Edinburgh, Seoul and Fortaleza amendments) [28] and the laws and regulations of the country. The protocol has been written, and the trial will be conducted according to the principles of ICH Harmonized Tripartite Guideline for Good Clinical Practice [26]. Patient data in the eCRF will be pseudonymised.

#### Benefit-risk assessment

Benefit-risk assessment was critically reviewed. Potentially, early stoma closure could result in more clinically relevant leaks of the rectal anastomosis as the time period for complete anastomotic healing is shorter. In order to exclude or minimize the risk of developing a clinically relevant leak of the rectal anastomosis after stoma closure the appropriate investigations confirming a healed anastomosis, which are usually done before late stoma closure, are mandatory also for early stoma closure. Several studies, including a large randomized controlled study, have not shown an increased risk for symptomatic leaks of the rectal anastomosis after early stoma closure [6, 8, 11]. Healing of the small bowel anastomosis after stoma closure may be impaired in the early group, because tissues may still be more friable such a short time after laparotomy. Again, several studies, including a large randomized controlled study, have not shown an increased complication risk for patients undergoing early stoma closure. On the other hand stoma closure may be more easy and faster as scaring may not yet be so pronounced less than ten days after stoma creation. Moreover, multiple complications may occur after stoma placement (e.g. kidney failure, prolapse, etc.), which may be minimized by early stoma closure. Finally, early stoma closure may have an impact on CoC potentially resulting in more patients receiving the full or at least a higher dose of scheduled chemotherapy which in turn could improve the oncological prognosis. Several studies including an analysis of our own data within a randomized controlled trial have shown that incomplete chemotherapy cycles and reduced dosages influence oncological outcome [14, 29, 30]. Therefore, even if a small to moderate increase of complications occurred due to early stoma closure this may be acceptable in order to improve overall outcome of patients.

## Discussion

Whether the time point of stoma closure influences the complete administration of the planned adjuvant chemotherapy, in patients with locally advanced rectal cancer undergoing LAR with TME after neoadjuvant therapy, remains unclear. Besides local complications stoma can have a negative impact on the patient’s psychological state since the mental health score was shown to be impaired in the presence of a stoma [9]. After tumour resection, stoma has a significant influence on the patients’ well being [9], affects them emotionally [8] and thus has a major impact on their compliance with the treatment. Some patients insist on stoma closure before the end of adjuvant chemotherapy cycles which may lead to a delay of the remaining therapy and may also increase complication rates [7]. Other patients stop therapy prematurely due to presence of a stoma [31].

That early stoma closure is an important issue in the complex therapy of rectal cancer patients is confirmed also by a newly started prospectively randomised Scandinavian multicentre study [32]. It compares early closure versus standard treatment (8–13 days vs. 12–26 weeks after surgery) with postsurgical morbidity as primary endpoint, QoL and the socio-economic effects of early closure as secondary endpoints. But this trial does not investigate CoC which therefore will remain an open question with high clinical relevance for the individual patient.

It is of major importance that patients undergo all chemotherapy cycles in order to maximally benefit from adjuvant treatment [30] but several studies show that not all patients are able to receive all cycles of the planned adjuvant therapy [14, 29, 30]. This may be due to suboptimal compliance with the therapy which can be explained by chemotherapy toxicity and postoperative complications.

Since early stoma closure is possible without increase in preoperative mortality and the complete administration of the planned chemotherapy cycles potentially improves the oncological outcome, it remains to be determined how early stoma closure influences CoC. This is the primary goal of the CoCStom trial and will contribute to clarifying how the largest benefit from adjuvant therapy can be gained for the affected patients. Therefore, CoC is a suitable primary endpoint and has high clinical relevance for the individual patient.

The trial may also have relevant socio-economic impact as early closure, if shown as equivalent or even superior, can cut costs due to reduced need for stoma care products and also due to a shorter overall period of hospitalisation.

CoCStom is a combined surgical-oncological trial. Its success requires a very good collaboration between surgeons and oncologists and the results may also have an impact on current guidelines as early closure of a stoma is currently not regarded as standard of care. Furthermore, results may also influence the “German DRG-System” on which is based the financing of medical in-house procedures. Presently, ileostomy closure within the same hospital stay as the rectal resection is not additionally reimbursed in contrast to late stoma closure in a second hospital stay.

### Trial status

Recruitment has started in December 2013. As of August 28^th^, 2015, 75 patients have been randomized.

## Additional files

Additional file 1:
**Trial visits in Arm A.** Example of visits and documented parameters for chemotherapy regimen with eight cycles. (PDF 17 kb) Additional file 2:
**Trial visits in Arm B.** Example of visits and documented parameters for chemotherapy regimen with eight cycles. (PDF 17 kb) Additional file 3:
**Secondary outcomes Definitions of secondary outcomes.** (PDF 21 kb) Additional file 4:
**Local ethics committees.** List of all ethics committees which approved the study protocol. (PDF 14 kb)
