# Supplementary material for: CoCStom trial: study protocol for a randomised trial comparing completeness of adjuvant chemotherapy after early versus late diverting stoma closure in low anterior resection for rectal cancer
Source: BMC Cancer. 2015 Nov 21;15:923. doi: 10.1186/s12885-015-1838-0 (PMC4654836; doi:10.1186/s12885-015-1838-0)
Supplement: Additional file 1: — Trial visits in Arm A. Example of visits and documented parameters for chemotherapy regimen with eight cycles. (PDF 17 kb) [file 12885_2015_1838_MOESM1_ESM.pdf]

Additional file 1.

Example of visits and documented parameters for chemotherapy regimen with eight cycles. Arm A.

**Group A:** **Early closure**	**CH: V1**	**CH: V2**	**CH: V3**	**ONK: V4**	**ONK: V5**
	Screening	Stoma closure	Day of discharge	CTx **cy 1** 4-12 W*	CTx **cy 2**
Demographic and clinical baseline data	**X**				
Inclusion/exclusion	**X**				
QoL	**X**			**X**	
Randomisation	**X**				
**Intervention**		**X**			
Secondary endpoints		**X**	**X**	**X**	**X**

	**ONK: V6**	**ONK: V7**	**ONK: V8**	**ONK: V9**	**ONK: V10**	**ONK: V11**	**K**	**CH: V12**	**CH: V13**
	CTx **cy 3**	CTx **cy 4**	CTx **cy 5**	CTx **cy 6**	CTx **cy7**	CTx **cy 8**		End of therapy 28 W*	End of follow-up 24 M*
**CoC(prim. endpoint)**								**X**	
QoL			**X**				**X**	**X**	**X**
Secondary endpoints	**X**	**X**	**X**	**X**	**X**	**X**	**X**	**X**	**X**

d: day; M: months; W: weeks; CoC: completeness of chemotherapy; CTx: chemotherapy; cy: cycle; * after randomisation, QoL: Quality of Life;

CH: V: visits in surgical department, **ONK: V**: visits in oncological department;

K: assessment of QoL at control visit (completion of the questionnaire ~4 weeks after application of the last CTx dose: hand out questionnaire at control visit or by mail).
