# Supplementary material for: CoCStom trial: study protocol for a randomised trial comparing completeness of adjuvant chemotherapy after early versus late diverting stoma closure in low anterior resection for rectal cancer
Source: BMC Cancer. 2015 Nov 21;15:923. doi: 10.1186/s12885-015-1838-0 (PMC4654836; doi:10.1186/s12885-015-1838-0)
Supplement: Additional file 2: — Trial visits in Arm B. Example of visits and documented parameters for chemotherapy regimen with eight cycles. (PDF 17 kb) [file 12885_2015_1838_MOESM2_ESM.pdf]

Additional file 2.

Example of visits and documented parameters for chemotherapy regimen with eight cycles. Arm B.

**Group B: Late closure**	**CH: V1**	**ONK: V2**	**ONK: V3**	**ONK: V4**	**ONK: V5**	**ONK: V6**
	Screening	CTx **cy 1** 4-12 W*	CTx **cy 2**	CTx **cy 3**	CTx **cy 4**	CTx **cy 5**
Demographic and clinical baseline data	**X**					
Inclusion/exclusion	**X**					
QoL	**X**	**X**				**X**
Randomisation	**X**					
Secondary endpoints		**X**	**X**	**X**	**X**	**X**

	**ONK: V7**	**ONK: V8**	**ONK: V9**	**K**	**CH: V10**	**CH: V11**	**CH: V12**	**CH: V13**
	CTx **cy 6**	CTx **cy 7**	CTx **cy 8**		Stoma closure	Day of Discharge	End of therapy 28 W*	End of follow-up 24 M*
**Intervention**					**X**			
**CoC(prim. endpoint)**							**X**	
QoL				**X**			**X**	**X**
Secondary endpoints	**X**	**X**	**X**		**X**	**X**	**X**	**X**

d: day; M: months; W: weeks; CoC: completeness of chemotherapy; CTx: chemotherapy; cy: cycle; * after randomisation, QoL: Quality of Life;

CH: V: visits in surgical department, **ONK: V**: visits in oncological department;

K: assessment of QoL at control visit (completion of the questionnaire ~4 weeks after application of the last CTx dose: hand out questionnaire at control visit or by mail)
