# Supplementary material for: CoCStom trial: study protocol for a randomised trial comparing completeness of adjuvant chemotherapy after early versus late diverting stoma closure in low anterior resection for rectal cancer
Source: BMC Cancer. 2015 Nov 21;15:923. doi: 10.1186/s12885-015-1838-0 (PMC4654836; doi:10.1186/s12885-015-1838-0)
Supplement: Additional file 3: — Secondary outcomes Definitions of secondary outcomes. (PDF 21 kb) [file 12885_2015_1838_MOESM3_ESM.pdf]

Additional file 3.

Definitions of secondary outcomes

**Secondary outcomes**	**definition**
Changes in QoL up to 24 months after randomisation	QoL baseline will be measured at randomisation (visit 1). Additionally it will be assessed for **group A (early closure)** at visit 4; at visit 6 or 8 (dependent on cycles number of each regimen: 4, 5 or 8 cycles); at control visit after visit 7, 8 or 11 (dependent on cycles number of each regimen: 4, 5 or 8 cycles) and at visit 12 and 13. For **group B (late closure)** QoL will be additionally assessed at visit 2; at visit 4 or 6 (dependent on cycles number of each regimen: 4, 5 or 8 cycles); at control visit after visit 5, 6 or 9 (dependent on cycles number of each regimen: 4, 5 or 8 cycles), and at visits 12 and 13. To determine QoL the EORTC QLQ-C30 and the CR 29 self-administered questionnaires, both validated in German, will be used. The specific items of interest are overall quality of life and those items related to stoma issues.
Stoma-related complications	Rate of stoma-related complications is defined as the percentage of patients developing stoma-related complications in relation to all patients with stoma.
Individual CoC rate	Individual CoC rate is the proportion of completed chemotherapy cycles calculated for each patient in relation to the planned number of cycles, determined 7 months after randomisation.
Percentage of patients stopping adjuvant therapy or undergoing dose modification or delay	Dose modification or delay measured 7 months after randomisation and defined as the proportion of randomised patients receiving a dose modification or delay in relation to the total number of randomised patients.
Toxicity of adjuvant chemotherapy per cycle	Toxicity of adjuvant chemotherapy will be assessed, per cycle, according to NCI-CTCAE v.4.03 criteria [1].
Disease free survival	Disease-free survival, determined from the date of randomisation to the date of diagnosis of recurrence or death during the 24 months follow-up period.
Local and distant recurrence-free survival	Local recurrence-free survival and distant recurrence-free survival, determined from the date of randomisation to the date of the respective recurrence event or death during the 24 months follow-up period.

Rate of symptomatic anastomotic leaks after stoma closure	Rate of symptomatic rectal anastomotic leaks (according to consensus definition)[2] is defined as the proportion of anastomotic leakages clinically symptomatic in relation to the total number of stoma closures within 30 days after the procedure.
Mortality	Mortality is determined from the date of randomisation to the date of death during 24 months follow-up period.
Postoperative complications (Clavien-Dindo Classification Grade 3 and 4 [3])	Number of re-operations is defined as the number of additional operations performed because of complications related to the stoma itself or the stoma closure procedure.
Estimation of economic impact by analysis of cumulative days of hospitalisation and number of readmissions	Cumulative days of hospitalisation are defined as the additional days in hospital over a period of 28 weeks after randomisation. Cumulative readmissions are defined as the number of readmissions due to complications related to the stoma closure operation or stoma-related complications over a period of 28 weeks after randomisation.

1. National Institutes of Health. NCI-CTCAE, v4.03. 2010. http://evs.nci.nih.gov/ftp1/CTCAE/CTCAE_4.03_2010-06-14_QuickReference_8.5x11.pdf. Accessed 01 Aug 2015.
2. Rahbari NN, Weitz J, Hohenberger W, Heald RJ, Moran B, Ulrich A, Holm T, Wong WD, Tiret E, Moriya Y *et al*. Definition and grading of anastomotic leakage following anterior resection of the rectum: a proposal by the International Study Group of Rectal Cancer. Surgery. 2011;147(3):339-351.
3. Dindo D, Demartines N, Clavien PA. Classification of surgical complications: a new proposal with evaluation in a cohort of 6336 patients and results of a survey. Ann Surg. 2004; 240(2):205-213.
