# Supplementary material for: CoCStom trial: study protocol for a randomised trial comparing completeness of adjuvant chemotherapy after early versus late diverting stoma closure in low anterior resection for rectal cancer
Source: BMC Cancer. 2015 Nov 21;15:923. doi: 10.1186/s12885-015-1838-0 (PMC4654836; doi:10.1186/s12885-015-1838-0)
Supplement: Additional file 4: — Local ethics committees. List of all ethics committees which approved the study protocol. (PDF 14 kb) [file 12885_2015_1838_MOESM4_ESM.pdf]

Additional file 4

List of ethics committees which approved the study protocol

1	Ethics Committee II of the Medical Faculty of Mannheim / University of Heidelberg
2	Ethics Committee of the Technical University Dresden
3	Ethics Committee of the Saarland State Chamber of Physicians
4	Ethics Committee of the University of Lübeck
5	Ethics Committee of the Hamburg Chamber of Physicians
6	Ethics Committee of the Medical Faculty of the University of Greifswald
7	Ethics Committee of the Medical Faculty of the University of Heidelberg
8	Ethics Committee of the Ludwig-Maximilians-University of Munich
9	Ethics Committee of the Medical Association North-Rhine
10	Ethics-Commission of the Medical Center-University of Freiburg
11	Ethics Committee of the Medical Faculty of the Eberhard-Karls-University and the University Hospital Tübingen
12	Ethics Committee of the Ulm University
13	Ethics Committee of the Medical Association Westphalia Lippe and the Medical Faculty of the Westphalian Wilhelms-University Münster
14	Ethics Committee of the Witten/Herdecke University
15	Ethics Committee of the State Chamber of Physicians Baden-Wuerttemberg
16	Ethics Committee of the Bavarian State Chamber of Physicians
17	Ethics Committee of the State Chamber of Physicians Brandenburg
18	Ethics Committee of the Chamber of Physicians in Bremen
19	Ethics Committee of the Friedrich-Alexander-University Erlangen-Nürnberg
20	Ethics Committee of the Medical Faculty of the University of Rostock
21	Ethics Committee of the State Chamber of Physicians of Rhineland-Palatinate
